# Pioglitazone in spontaneous subarachnoid hemorrhage: study protocol of a multicenter, double-blind, randomized trial (PSSH)

**DOI:** 10.3389/fphar.2023.1323292

**Published:** 2024-01-05

**Authors:** Junhui Chen, Mingchang Li, Lei Chen, Qinyi Xu, Tengfeng Yan, Chunlei Zhang, Ping Hu, Jianqing He, Xun Zhu, Xingen Zhu, Yuhai Wang

**Affiliations:** ^1^ Department of Neurosurgery, 904^th^ Hospital of Joint Logistic Support Force of PLA, Wuxi Clinical College of Anhui Medical University, Wuxi, China; ^2^ Department of Neurosurgery, Renmin Hospital of Wuhan University, Wuhan, China; ^3^ Department of Human Anatomy and Neurobiology, School of Basic Medical Science, Central South University, Changsha, Hunan Province, China; ^4^ Department of Neurosurgery, Wuxi Huishan Peoples Hospital, Wuxi, Jiangsu, China; ^5^ Department of Neurosurgery, The Second Affiliated Hospital of Nanchang University, Nanchang, China; ^6^ Department of Neurosurgery, The Second Hospital of Tianjin Medical University, Tianjin, China

**Keywords:** clinical trial, pioglitazone, SAH, PPARγ, neuroprotection

## Abstract

**Introduction:** Spontaneous subarachnoid hemorrhage (SAH), is a disorder that may be fatal and is primarily caused by a ruptured brain aneurysm. Despite significant leaps forward in the methods to produce aneurysms, the long-term outcomes did not much improve. Pioglitazone is a medication that has been authorized by the FDA as an agonist for the peroxisome proliferator-activated receptor-gamma (PPARγ). Pioglitazone or PPARγ has neuroprotective benefits in animal experiments both during and after traumatic brain injury (TBI) and SAH. Nevertheless, the treatment impact of Pioglitazone on humans is still unknown at this time. As a result, we will conduct a randomized, double-blind, placebo-controlled trial to explore the impact of pioglitazone on SAH.

**Methods/Design:** This trial will recruit 400 patients with SAH from four Chinese hospitals. These patients will be equally and randomly assigned to Pioglitazone and placebo control groups for up to 30 days. Scores on the modified Rankin scale (mRS) are the primary outcomes. The secondary outcomes are a 30-day all-cause mortality rate, 6 months of Montreal cognitive assessment (Mo-CA), delayed cerebral ischemia, the requirement for intensive care, the incidence of sepsis, *etc*. All serious adverse events (SAEs) were recorded during the hospital. Every primary and safety analysis was conducted based on the intention-to-treat technique. The participants were given either a matching placebo or 15 mg of pioglitazone, with dose titrated to a target of 45 mg daily. Data on the therapeutic use of pioglitazone after SAH will be provided as a consequence of the findings of this experiment. In addition, this pilot trial is the first to prospectively investigate the effectiveness and safety of pioglitazone in patients with SAH.

**Ethics and dissemination:** Ethics approval was obtained from the Medical Ethics Committee of 904th Hospital of Joint Logistic Support Force of PLA (Wuxi Taihu Hospital, approval No. 20220701). The findings of the trial will be presented at conferences, discussed in relevant patient groups, and published in peer-reviewed journals.

**Clinical Trial Registration:** clinicaltrials.gov, identifier ChiCTR2200062954.

## Introduction

Subarachnoid hemorrhage (SAH) is a condition that often occurs in people who have cerebral vascular disease and is hallmarked by increased morbidity and mortality rates and a poor prognosis, particularly in patients who have hypertension. There have been reports indicating that the incidence rate of SAH ranges from 6.2 to 10 cases per 100,000 people in the West (Korja et al., 2016; Mackey et al., 2016; Etminan et al., 2019) and affects approximately 600 000 patients worldwide (Feigin et al., 2009). SAH is associated with a high overall mortality rate that may reach as high as 67% (Nieuwkamp et al., 2009), and only a third of survivors remaining live dependently (van Gijn et al., 2007). Since SAH results in a significant loss of production and resources, it places a considerable cost on society (Zolnourian et al., 2020).

Many previous scholars have attempted to improve outcomes after SAH, and statins (Kirkpatrick et al., 2014; Naraoka et al., 2018), magnesium (Dorhout Mees et al., 2012), and tranexamic acid (Post et al., 2021) are among the medications that have demonstrated superior neuroprotective benefits in basic research; nonetheless, these efforts to enhance outcomes after SAH have primarily been unsuccessful. Although numerous studies have shown that statins or clazosentan may significantly alleviate cerebral vasospasm (CVS), these medications have little impact on the outcomes after a SAH (Macdonald et al., 2011; Chen J. et al., 2020). Despite significant advancements in methods for producing aneurysms, long-term outcomes have not been improved, and there is a lack of medical therapy that is suited for the condition.

Red blood cells and lysis products in subarachnoid space or brain tissue may produce a significant inflammatory response along with a rise in levels of oxyhemoglobin, endothelin, nitric oxide, and proinflammatory cytokines, resulting in early brain injury (EBI) and CVS and ultimately leading to neurological dysfunction and death after SAH (van Gijn and Rinkel, 2001; Chen et al., 2016; Ch et al., 2018; Chen et al., 2019a; Chen J-H. et al., 2020). Therefore, finding a way to prevent CVS and EBI should be the primary objective of any novel pharmacological therapy for SAH. Pioglitazone is a drug that has been approved by the FDA as a peroxisome proliferator-activated receptor-gamma (PPARγ) agonist. PPARγ is a nuclear hormone receptor, and its activation regulates many aspects of inflammatory processes as well as mitochondrial homeostasis. PPAR-γ agonists can block the generation of pro-inflammatory mediators and nitric oxide by inhibiting the activation of astrocytes and microglia (Storer et al., 2005); Additionally, Oxidative stress and mitochondrial biogenesis are regulated by the transcriptional factor, PPAR-γ coactivator 1-α (PGC-1α) (Eschbach et al., 2015). Animal studies have proven that Pioglitazone or PPAR-γ exerts neuroprotective benefits in multiple disorders of the central nervous system (CNS), however, the specific neuroprotection pathways of PPAR-γ agonists/Pioglitazone are still obscure (Zuhayra et al., 2011; Pilipović et al., 2015; Liang et al., 2022). Animal models of Parkinson’s disease treated with a dosage of pioglitazone equivalent of the human FDA-approved dosage demonstrated both effective CNS penetration of pioglitazone and neuroprotection benefits (Kernan et al., 2016; Pinto et al., 2016). Nevertheless, it is yet unknown whether or not Pioglitazone has any neuroprotective benefits following SAH in humans.

Thus, the focus of the proposed clinical study is to examine the effectiveness and safety of pioglitazone in treating SAH.

## Methods/design

### Study design

This multicenter, double-blind, placebo-controlled, parallel-group, randomized clinical trial will be conducted in the 904^th^ Hospital of Joint Logistic Support Force of PLA (Wuxi Taihu Hospital), Renmin Hospital of Wuhan University, The Second Affiliated Hospital of Tianjin Medical University, The Second Affiliated Hospital of Nanchang University.

### Patient and public participation

Patient and public involvement Patients and/or the public did not participate in any stage of the design, execution, reporting, or distribution of the plans of this study.

### Ethical considerations and informed consent

The researcher or other authorized personnel will obtain written informed permission from each patient or respective family member or guardian after explaining the benefits and risks of participating in the research. This trial has received the written approval of the Medical Ethics Committee of 904^th^ Hospital of Joint Logistic Support Force of PLA (Wuxi Taihu Hospital, No. 20220701), and other Sub-centers. The principles outlined in the Declaration of Helsinki, which were developed by the World Medical Association, have been adhered to throughout the execution of this project.

### Qualifications for participating centers

The researchers will cooperate with four medical centers in various parts of China (north, south, east, and central China). Each facility is equipped with over two referral units and has a skilled treatment team who has experience in diagnosing SAH and providing effective treatment. As a result, it is generally accepted that this cohort is capable of providing an accurate representation of the population at both the regional and national levels. All participants in this trial will be monitored and treated at one of the collaborating medical centers throughout the study.

### Study participants

Participants for this research will be selected from SAH patients currently being treated at any of the four hospitals. Participants will be recruited consecutively across all of the clinical facilities. The patients will be selected using specified eligibility requirements, and then the competent authority will randomly assign them to one of the treatment groups.

#### Inclusion criteria

To be eligible for inclusion in the study, patients must satisfy all of the following conditions.

1. Ranging in age from 18 to 70 years
2. Head CT evidence of SAH.
3. CTA, MRA, or DSA confirmed the presence of an intracranial aneurysm as the cause of the patient’s recent SAH.
4. The time between SAH onset and admission should not exceed 24 h.
5. The patient’s caregiver must agree to the research protocol and sign an informed consent form as per the guidelines set out by the ethics committee.

#### Exclusion criteria

1. Symptoms consistent with SAH lasting more than 3 days before admission
2. The CT scan shows a pattern of hemorrhage consistent with a traumatic SAH.
3. Patients addicted to drugs
4. Patients with chronic renal failure that requires treatment with dialysis
5. Patients with a life expectancy of fewer than 6 months due to a severe terminal illness
6. Severely coagulation dysfunction patients
7. Patients with organ failure
8. Individuals suffering from severe psychosomatic illness
9. Pregnant or lactating women
10. The caregiver responsible for the patient declined to provide permission for the patient to take part in this experiment.
11. The patient was enrolled in a different randomized controlled trial (RCT) aimed at treating SAH.
12. Patients who had taken rosiglitazone or pioglitazone within the previous 90 days prior to randomization
13. Current participation in a conflicting clinical trial. A conflicting clinical trial is defined as a trial with any of the following:
  (1) Intervention that is known to affect the incidence of stroke or myocardial infarction
  (2) An experimental pharmacological intervention
  (3) Stroke as a possible outcome
  (4) Exclusion for participation in another trial
14. Clinically significant structural brain disease that the investigator believes would interfere with study evaluations
15. Symptoms of congestive heart failure in the past
16. Known intolerance to rosiglitazone or pioglitazone
17. Diabetes Mellitus, Type I or II
18. Previous Bladder Cancer History
19. Evidence of a previous case of macular edema
20. Individuals subject to temporary exclusions are eligible for enrollment after their status has stabilized. The temporal exclusions include those with:
  (1) Over 2.5 times the standard upper limit for alanine aminotransferase (ALT)
  (2) Below 8.5 g/dL of hemoglobin
  (3) Pitting edema of the feet and legs, mild to severe (IRIS grade 3 or 4)
  (4) Scheduled carotid surgery or carotid stenting (delaying randomization till 2 weeks after the operation)

### Randomization

A statistician will use a random number generator to assign a number to each of the 400 participants based on the time they were admitted. The participants will be randomized to either the pioglitazone group or a placebo control group.

### Blinding and emergency unblinding

The treatment plan that will be administered to each participant will be developed using a randomized allocation sequence and then put in numbered, opaque envelopes that have been sealed. The blinded codes will be preserved by the drug administrator. In the case that a patient has a severe adverse reaction and it is essential to promptly establish the drug that was administered, a primary investigator from the research unit will open the envelope containing that patient’s information. The participation of the patient in the experiment will be discontinued as soon as the data have been unmasked. The findings will be communicated to the clinical research associate. The unmasking will be documented, along with the cause for it and the date it took place, and the researchers will sign the case report form.

### Interventions

**FIGURE 1:**
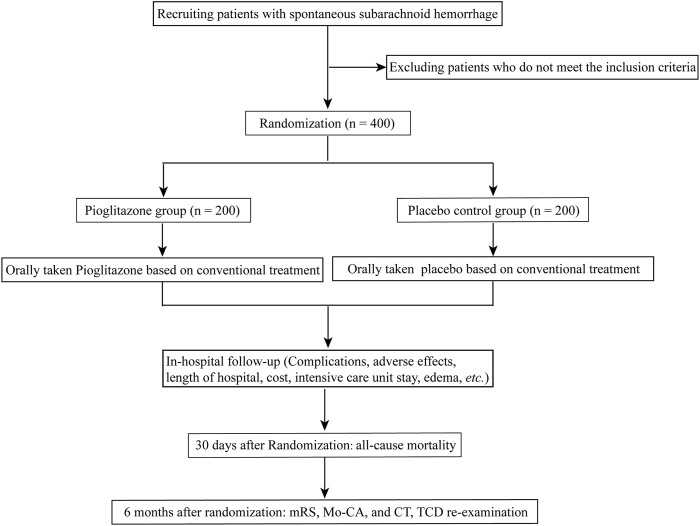
Flow chart of the trial.

Patients in both groups will be given standard therapies, which may include sedation, absolute bed rest, maintaining fluid balance, and hemostasis. Pioglitazone group: Patients will also be given an oral dosage of pioglitazone (approval No. GYZZ J20090134; Tianjin Taketian Pharmaceutical Co. LTD, Tianjin, China) in addition to standard care. Patients were allocated to receive a starting dosage of 15 mg of pioglitazone titrated to reach a targeted dosage of 45 mg daily. Placebo control group: patients will get placebos orally (starch tablets with the same appearance as atorvastatin) in addition to their standard therapies. The recommended dose will be the same as for pioglitazone. The trial’s workflow is depicted in Figure 1.

### Procedures

At admission to the hospital, all patients underwent baseline cranial CT and CT angiography (CTA) or DSA to confirm the diagnosis of SAH. Then, investigators recorded sex, age, Fisher Grade Score, Glasgow Coma Scale, Hunt-Hess Scores, *etc.* for each patient. The investigators completed the enrollment assessment and random allocation as soon as possible. Following random assignment, those in the intervention group were started on the therapy immediately.

### Outcome measures

#### Primary outcomes

Clinical outcome at 6 months and 12 months post-randomization is the primary endpoint measure evaluated using the modified Rankin Scale (mRS) through a standardized interview (Wilson et al., 2005). Research physicians blinded to treatment assignment performed all evaluations. Medical doctors conducting research were instructed on how to undertake the structured face-to-face interview to calculate the mRS score as per standard operating procedures (SOPs). Clinical outcomes were classified as good (mRS 0–3) or poor (mRS 4–6) after the scores were recorded.

#### mRS scores

0 - no symptoms, 1 - no significant disability, carrying out all usual activities normally while experiencing some symptoms, 2 - slight disability, self-sufficient in taking care of basic needs but without the skills to complete all previous activities, 3 - moderate disability, requires some help, but able to walk unassisted, 4 - moderately severe disability, incapable of attending to own bodily needs without assistance, and unable to walk unassisted, 5 - severe disability, needs constant nursing care and attention, incontinent, and bedridden, 6 - deceased.

#### Secondary outcomes

The secondary outcome measures were all-cause mortality at 30 days, 6 months Montreal cognitive assessment (Mo-CA), delayed cerebral ischemia, the serum PPAR- γ levels at 1, 3 and 7 days after operation. duration of hospitalization, pulmonary and cardiovascular complications, rebleeding, intensive care unit stay, the incidence of sepsis, the occurrence of hydrocephalus, and treatment modality used to treat the hydrocephalus.

#### Mo-CA

Long-term cognitive function is assessed with the Montreal cognitive assessment (MoCA) (Ozdilek and Kenangil, 2014). The MoCA is rated on a scale ranging from 0 to 30 points. The MoCA threshold of 26 points is used to diagnose cognitive impairment and a score of less than 22 is considered to indicate serious cognitive impairment (the optimum threshold score for cognitive domain impairments according to the MoCA) (Wong et al., 2015).

### Follow-up

The follow-up duration will be 6 months and 12 months beginning from the time when patients were randomized. Patients will be contacted by phone and asked to come back to the hospital for further examinations, which will include conventional CT scans, transcranial Doppler ultrasonography, and neurological scale evaluations. If a patient cannot be located for follow-up, the most recent data evaluated will be used to determine the patient’s values for the 6-month follow-up (Table 1).

**TABLE 1:** Outcome measures and measurement points.

Visit number	Screening period	Follow-up period		
	1	2	3	4
Research arrangement	Screening	In-hospital	30 days	6 months
Informed consent	√			
Selection criteria	√			
Baseline information	√			
General history	√			
Symptoms and physical examination	√			
GCS grading	√			
WFNS grading	√			
Hunt-Hess grading	√			
Conventional brain CT	√	√		
Transcranial Doppler	√	√		
Electrocardiogram	√	√		
Blood sugar	√	√		
Blood NT-proBNP	√	√		
All-cause mortality			√	
mRS score			√	√
Mo-CA			√	√
End point evaluation		√	√	√
Adverse event evaluation		√	√	√
Complications		√	√	√
Intensive care unit stay		√	√	√
Length of hospital		√	√	√
Hospitalization costs		√	√	√

### Safety and serious adverse events

The following are examples of potential adverse reactions: hyperkalemia, gastrointestinal dysfunction, immune system dysfunction, insomnia, serum glucose disruption, pruritus, pitting edema of the feet or legs, and abnormalities in serum transaminase and phosphocreatine kinase. To make it easier for patients to record accurately the shifts in their illnesses that occurred as a result of drug usage, the doctors will avoid asking patients leading questions. In addition to the therapeutic outcomes, any adverse events or unforeseen adverse reactions (signs, symptoms, and lab tests) will be noted. If there is a serious adverse event that takes place, the researchers will quickly implement the necessary treatment procedures. The drug will be withdrawn. The time of drug withdrawal, time of onset, length of symptoms, response to therapy, and the final result will all be documented. Within 24 h, the researchers will notify the ethics committee, the Drug Supervision and Administration Department, and any other applicable administrative agencies of any serious adverse events. The report will be signed and dated by the researchers.

### Data monitoring body and management

This research will be carried out in compliance with the guidelines for good clinical practice as well as the principles outlined in the Declaration of Helsinki. An independent clinical trial data management committee (CTDMC) composed of members of the ethics committee, neurosurgeons, physicians, information specialists, and intensivists will complete non-blinded evaluations of all of the patients’ effectiveness and safety data independently, comprising AEs and dropout rates, recorded from the time of informed consent to 180 days after the randomization process. The CTDMC is responsible for the examination and analysis of clinical data and holds regular meetings to control the quality of the trials.

The patient’s health records will be digitized and stored on a computer, and the data will be uploaded to the Electronic Data Capture system (ResMan, www.medresman.org). Details provided on the case data form will be checked for accuracy. Any changes or corrections made will retain a clear record of the original. When an amendment or correction is made, the researcher will note the date, sign their name, and provide an explanation (if required). All of the patient’s medical history, including any alterations, deletions, or updates, will be stored digitally. The audit trail will be secured in a way that prevents any unauthorized changes or deletions. The clinical data management system will have authority management such that only authorized personnel will be allowed to operate it. Electronic signatures are widely used in ERMs (electronic management systems). To ensure the privacy and security of the collected clinical data, only authorized users will be able to access the system and enter, modify, or view the data. All documentation shall be kept for at least 5 years following the conclusion of the experiment, as required by the China Drug Clinical Trial Management Specification.

### Trial quality assurance and control

Quality control and assurance will be handled by CTDMC. A clinical research associate who has been assigned by the sponsor will make regular hospital visits to check that the study is conducted according to plan and that all data are being collected accurately. Before, during, and after the experiment, the investigators will follow SOPs for clinical studies. Inspectors will check case registration forms to ensure accurate and complete information throughout the trial. Researchers will receive training that will standardize their data collection and evaluation practices. To guarantee accurate and trustworthy data, researchers will meticulously document all data on case report forms. Researchers will compare aberrant lab test findings to normal reference ranges. To guarantee that the inferences are valid and based on the original data, we will double-check all of our observations and findings. Relevant data management procedures will be implemented throughout the stages of clinical trials and data analysis. Active measures will be taken to control the dropout rate to within 20%.

### Sample size estimates

According to previous clinical data and our pilot study’s preliminary experiments, 51.9% (42/81, retrospective data) of patients in the untreated group had a good clinical outcome compared with 63.3% (19/30) in the intervention group. The decision to recruit 378 patients (189 in each arm) was based on a power analysis with an alpha of 0.05, a statistical power of 80%, and a 10% loss to follow-up. We decided to enroll 400 patients (200 in each arm). All of the data from the study’s baseline as well as its outcomes were input into the database by a research nurse. The analysis of the data was performed by the research coordinator. The CTDMC checked the data and discussed the findings.

### Statistical analysis

SPSS 20.0 will be utilized for the analysis of all the data. Measurement data will be expressed as the mean ± SD. After the first half of the patients were recruited, CTDMC evaluated the safety of the trial participants, efficacy, and overall progress of the study and did a masked interim analysis. All patients assigned a random number during the randomization process will be examined using an intention-to-treat analysis. If any measurements in the medical records or outcome measures are found to be incomplete during the statistical analysis, the final data obtained will be considered the final result for use in the statistical analysis.

Two-sample t-tests and odds ratios (OR) with 95% confidence intervals (CI) are provided to identify variations across treatment groups. The criterion for statistical significance will be set at *p* < 0.05. Adjusted odds ratios (aORs) will be calculated using multivariate logistic regression to account for the impact of the treatment facility and any variations in baseline variables for the primary and secondary outcomes. We also did per-protocol and as-treated analyses.

## Discussion

This protocol describes the PSSH study, which will be the first large sample multicenter project to prospectively examine the effectiveness and safety of Pioglitazone in patients with aSAH, from hospital admission to 6 months follow-up. If it succeeds, it might help patients with SAH in the long term, giving them hope despite their complicated neurocognitive symptoms and disabilities. It also has important clinical translational value.

In aSAH patients, neuroinflammation occurs shortly after the bleeding and is associated with EBI, delayed cerebral ischemia, poor functional outcome, and case fatality (Chen et al., 2021). Inhibiting microglial and astrocyte activation with PPAR-γ agonists reduces the release of pro-inflammatory mediators and nitric oxide (Storer et al., 2005). Our earlier experimental SAH investigation found that PPAR-γ upregulation after SAH may ameliorate neurological dysfunction and alleviate EBI (Chen et al., 2019b). As a result, PPAR-γ agonists (Pioglitazone) may emerge as a safe and successful means of treating aSAH. Pioglitazone may be an important target to reduce EBI and improve the long-term outcome after aSAH.

Pioglitazone’s most frequent side effects are dosage-dependent and include nausea, vomiting, edema, and myopathy, all of which improve after the medicine has stopped being taken by the patient. Pioglitazone has a low incidence of side effects, and it has an excellent safety and tolerability profile with no known safety concerns. (Dormandy et al., 2005; Belfort et al., 2006).

Nonetheless, we believe that a well-designed large sample multicenter trial could help us better understand the value of Pioglitazone in aSAH patients. Our study’s findings might have significant significance for patient care in clinical settings.
